# The Effect of Plate Working Length on Interfragmentary Movement in a Distal Femoral Fracture: A Biomechanical Study

**DOI:** 10.7759/cureus.94479

**Published:** 2025-10-13

**Authors:** Jacob Lagoni, Asger M Haugaard, Isabelle B Pfander, Ilija Ban, Marie S Traberg, Søren Ohrt-Nissen

**Affiliations:** 1 Orthopedics, Copenhagen University Hospital, Hvidovre, Copenhagen, DNK; 2 Orthopedics, Bispebjerg Hospital, Copenhagen, DNK; 3 Health Technology, Technical University of Denmark, Copenhagen, DNK; 4 Spine Unit, Department of Orthopedic Surgery, Rigshospitalet, Copenhagen, DNK

**Keywords:** biomechanical properties, distal femoral fracture, distal femur locking plate, fracture healing factors, working length

## Abstract

Background

Distal femoral fractures are typically treated by bridging with locking plates. The mechanical environment is influenced by working length (WL), with larger WL theoretically increasing the interfragmentary movement (IFM). However, considering the strength of modern locking plates, the effect of WL is questionable. The aim of this study was to quantify the effect of WL on IFM in distal femoral fractures at an approximated physiological load.

Materials and methods

Ten fourth-generation composite femurs with a 10 mm transverse fracture gap were uniformly fixed to a 13-hole locking plate with a short (95 mm) and long (175 mm) WL. The constructs were mounted in an Instron machine (Instron, Norwood, MA) and axially loaded from 50 to 750 N, 50,000 times. The micromotion was measured with the digital image correlation method of the implant-femur construct under cyclic loading. A simplified Bernoulli-Euler beam model analysis was performed to evaluate the contribution of plate deformation on the observed micromotion and serve as a control of the results.

Results

In the cyclic loading analysis, the median (min-max) IFM was 0.81 mm (0.66-1.73) and 1.64 mm (0.26-2.35), respectively, for the short and long WL group (p = 0.421). The median lateral movement (shear) was 0.89 mm (0.71-1.52) and 2.81 mm (2.16-3.94), respectively, in the short and long WL group (p = 0.007). The range of deformation remained constant throughout the 50,000 cycles, irrespective of the WL. In the beam model, we found the contribution of axial plate compression to the observed deformation was negligible. Using the beam model, we calculated an expected axial IFM of 0.82 mm for the short WL and 1.52 mm for the long WL.

Conclusion

During an approximated physiological loading, we found less than 1 mm difference in axial IFM between a short and long WL. We found substantially higher levels of shear movement in the long WL. We have provided a simple model to predict IFM based on WL and femur characteristics.

## Introduction

The healing of a distal femoral fracture is highly dependent on the balance between stability and biology at the fracture site. Surgical treatment can influence the mechanical environment, but the mechanisms are still poorly understood. Perren [1] proposed a strain theory in 1979, describing how the amount of interfragmentary movement (IFM) (or compressive strain) determines the subsequent differentiation of fracture gap tissue. This is based on the theory that the tissue cannot exist in an environment exceeding the strain tolerance of these tissues. One way to control the biomechanical environment and IFM is by choice of surgical technique and construct. Locking plates are considered the gold treatment for distal femoral fractures, functioning as a bridging osteosynthesis. This allows for stabilization of the fracture site, without stripping the bones’ blood supply during plate insertion. Several case reports [2] have been published on the use of locking plates for distal femoral fractures, and despite an increasing clinical experience with the technique, the rate of non-union remains relatively high between 10% and 20%.

One key element of the bridging technique is choosing the appropriate working length (WL), which is the distance between the two closest screws at either side of the fracture. Theoretically, increasing the WL will increase the potential IFM. The actual IFM will depend largely on the stiffness of the construct and the load that is applied to the leg in the postoperative phase. Previous biomechanical studies [3-10] vary greatly in both surgical construct and the load and number of repetitions during testing. It is not clear to what extent IFM is affected by WL using modern locking plates at a physiological load.

The aim of this study was to determine whether plate WL significantly alters IFM and shear displacement in distal femoral fractures under physiological loading. We conducted a series of mechanical experiments involving surrogate femoral specimens and distal femoral locking plate fixations under different WL configurations to investigate the characteristics of IFM at the fracture site.

## Materials and methods

Surgical specimens were 10 fourth-generation composite femurs composed of short fiber-filled epoxy simulating cortical bone, with a 17 PCF solid foam cancellous core. The bones have an overall length of 485 mm, with a 10 mm hollow central canal. The neck angle is 122 degrees, with an anteversion of 15 degrees. The 10 surrogate bones were divided into a short (95 mm) and long (175 mm) WL group.

**Figure 1:**
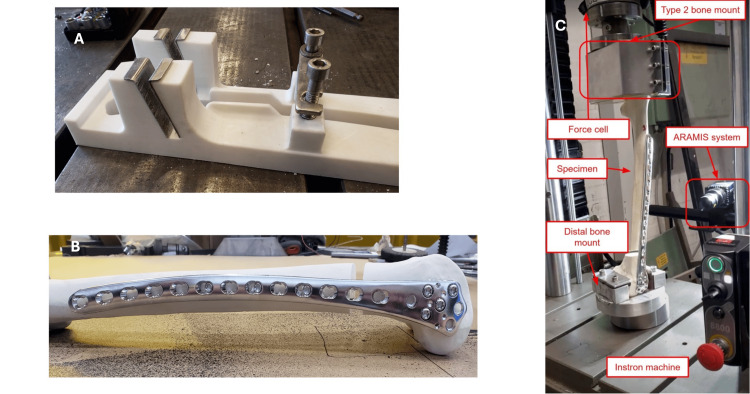
Mechanical test setup. (A) The 3D-printed mold for creating uniform osteotomies. (B) The final osteosynthesis, with a 10 mm fracture gap. (C) The setup for mechanical cyclic testing.

For surgical fixation, we used 316 mm long stainless steel 13-hole distal femur locking compression plates (LCP-DF 4.5/5.0, DePuy Synthes, Raynham, MA). Self-tapping locking screws with a diameter of 5.0 mm and a length of 36 mm were used along the femoral shaft, and locking screws with a length of 70 mm were used in the metaphysis. The plate was held in place with a 3D-printed fixture to uniformly apply the plate laterally to the bone with a short (95 mm) and long (175 mm) WL. After plate fixation, a template was used to create a 10 mm transverse fracture gap in the metaphysis (Figure 1A).

All plates were fixed with four distal and three proximal screws. All distal screws were fixed in the same holes and equally tightened. The three proximal screws were also equally tightened and applied in the same hole within each group (Figure 1B). Final tightening of locking screws was performed using a torque limiter set to 4.0 Nm per manual instructions.

Mechanical testing

The constructs were mounted in an Instron compression testing machine (Instron, Norwood, MA) and axially loaded from 50 to 750 Newton (N), 50,000 times (Figure 1C). To mount the constructs in the Instron machine used for cyclic loading, custom epoxy molds were created to hold the proximal end, and steel and aluminum mounts were used to hold the distal end.

The ARAMIS 12 M measuring system, which employs the digital image correlation (DIC) method, was used for data collection. Two Titanar B 75 mm lenses were used to give a measuring volume of 100 mm x 75 mm x 25 mm.

Theoretical beam modeling

**Figure 2:**
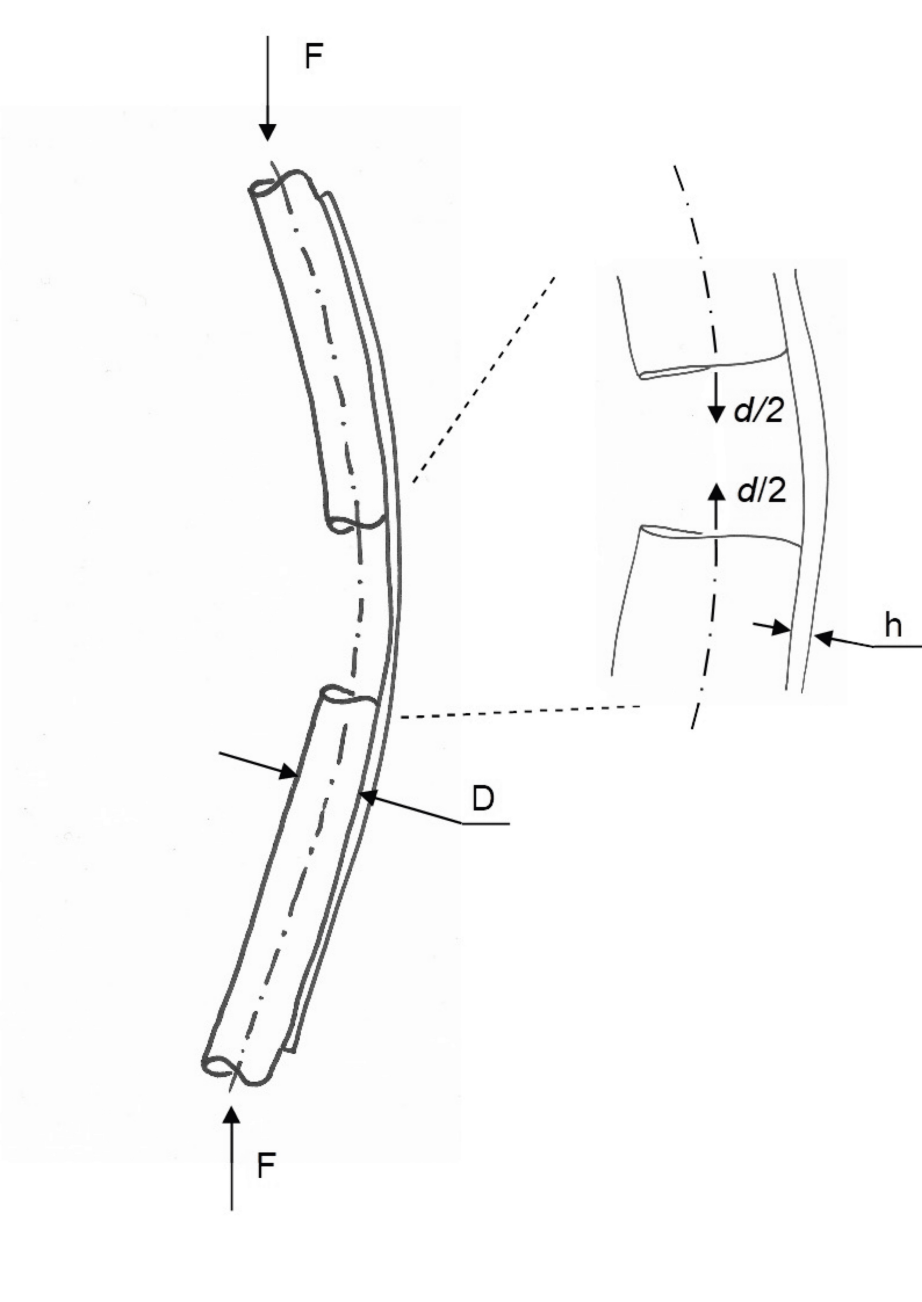
Bone-plate deformation. Schematic showing the bone/plate construct. The screws are not shown. The deformation is greatly exaggerated.

A theoretical beam model of the construct was developed based on Bernoulli-Euler beam theory [11]. The aim was to simplify the model to the point that it can be applied by clinicians in daily use. The bone is assumed cylindrical with diameter *D*. The bending moment relative to the neutral axis of the beam (plate) can be derived from an appropriate free-body diagram. At the level of the most distal screw in the proximal fragment, the moment arm is approximately *D/2*. Furthermore, due to the curvature of the lateral cortex of the femur, the force is approximately parallel to the plate. In other words, the resulting axial force *F* is assumed to act in the center of the bone and parallel to the beam (Figure 2).

The plate is assumed to be of uniform rectangular cross-section with thickness *h* and width *w*. The holes were neglected, and symmetry boundary conditions were applied. The second moment of inertia *I* of the plate cross-section is thus:

$I=\frac{{\rm wh}^3}{12}$

Symmetry boundary conditions are applied, so the plate is considered cantilevered in the middle of the fracture and modeled as two beams of length *L/2* each, where *L* is the working length. The angular deflection of a cantilevered beam of length *L/2* subjected to a bending moment *M* is:

${\Theta}=\frac{ML}{2EI}$​​​

Where *E* is the elastic modulus of the plate material. For steel, the elastic modulus is 210 GPa. The plate is orders of magnitude stiffer in axial compression than in bending, so this effect is ignored. The near-side (lateral side) deflection of the femur is thus by definition nil. Under the assumption of small angles, the deflection of the middle of the fracture gap is:

$\frac{d}{2}=\frac{\theta D}{2}$

Applying the above, the total micro-motion across the fracture gap is calculated as:

$d=\frac{6FD^2L}{Ewh^3}$​​

Alternatively, substituting *F* for mg, a patient of mass *m* (in kg) supporting only on the operated limb, as during the gait cycle, will cause the IFM.

$d=\frac{6mgD^2L}{Ewh^3}$

Statistical analysis

Statistical analyses were performed using R version 3.4.0 (R Foundation for Statistical Computing, Vienna, Austria). Data are reported as median with minimum-maximum values. The primary outcome was IFM after 50,000 cycles. We analyzed differences in IFM between the two groups using the Wilcoxon rank-sum test.

## Results

**Figure 3:**
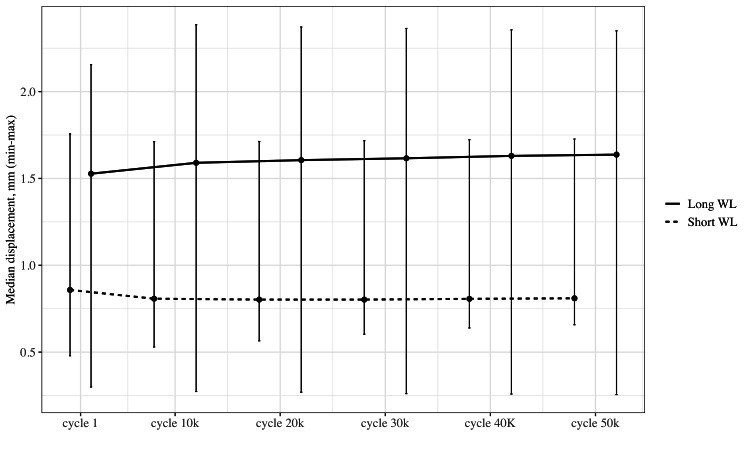
Compressive strain. Graph showing the median interfragmentary movement (IFM) in the long and short working length (WL) groups during cyclic loading.

In the cyclic loading test, the median IFM in the short WL group was 0.81 mm (min-max: 0.66-1.73) and 1.64 mm (min-max: 0.26-2.35) in the long WL group (p = 0.421) (Figure 3). We saw no cases of construct failure (collapse of the construct) during the 50,000 cycles.

**Figure 4:**
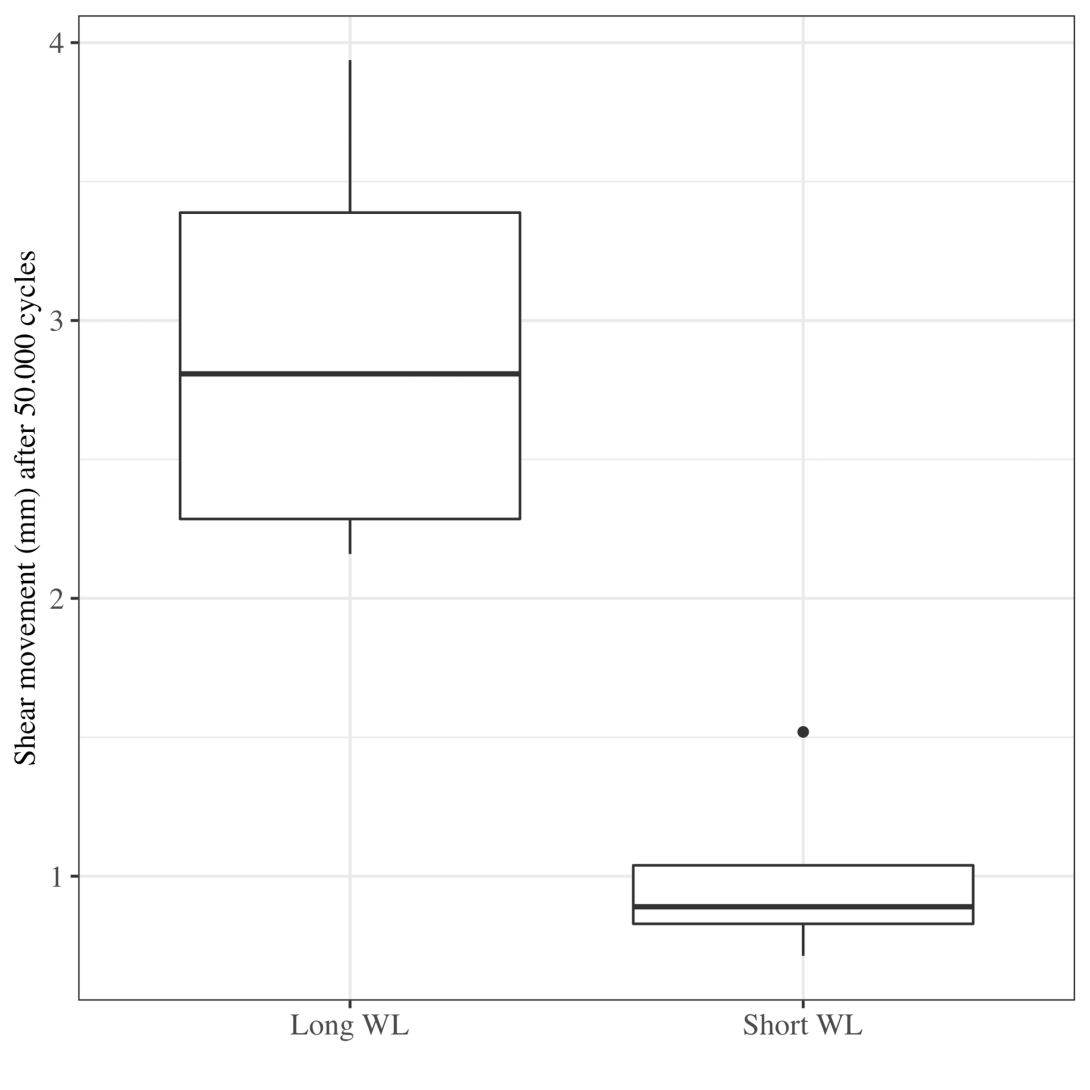
Shear movement. Graph showing the results for shear movement under compression with long and short working length (WL) after 50,000 cycles.

The median lateral movement (shear) after 50,000 cycles was 0.89 mm (0.71-1.52) in the short WL group and 2.81 mm (2.16-3.94) in the long WL group (p = 0.007) (Figure 4).

Beam model

In the simplified beam model, we found, firstly, that the axial compression forces were several orders of magnitude below the critical buckling load of the plate and, secondly, that the ranges of axial deflection from pure compression of the plate were 0.0013 mm (short WL) and 0.0024 mm (long WL). Thus, the contribution of axial plate deformation to the IFM was negligible.

We calculated an expected IFM based on the model using the following parameters: *F* = 700, *h* = 5.5 mm, *w* = 16 mm, *E* = 210 GPa, *D* = 34 mm, and *L* = 95 mm (short WL) or 175 mm (long WL). The model predicted:

$d = \text{IFM} = 0.82 \text{ mm (short WL)}$

$d = \text{IFM} = 1.52 \text{ mm (long WL)}$

**Figure 5:**
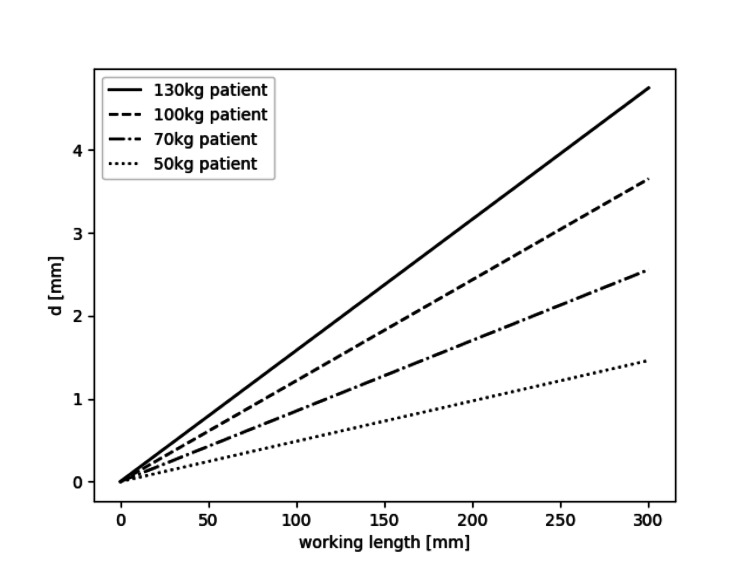
Prediction model. Model predictions of interfragmentary movement (IFM) (mm) of patients of varying mass, assuming that the patient is supporting their full weight on the operated limb. Graphs for various values of femur diameter, *D*, are reported. The elastic modulus is set to 210 GPa. Gravitational acceleration is set to the plate thickness and width are *h *= 5.5 mm and *w* = 16 mm, respectively. These parameters correspond to a standard distal femur locking compression plate.

Based on the equation, model predictions across a range of parameters were generated (Figure 5).

## Discussion

Distal femoral fractures often occur in patients who are elderly and frail. Surgical treatment should allow for immediate mobilization, and biomechanical studies should therefore attempt to replicate the physiological load after surgery to provide a meaningful estimate of the importance of biomechanical parameters. The current study shows that a substantial increase in WL resulted in an increase in IFM of 0.8 mm. We noted a higher variation between specimens in the WL group (Figure 3), indicating a less predictable IFM in the clinical setting. These experimental results correlate well with our Bernoulli-Euler beam model, by which we can estimate the expected deformation. This simple model has the potential to guide clinicians in predicting the IFM based on WL and femur characteristics (Figure 5).

We found that increasing the WL resulted in substantially higher shear movements (Figure 4) and much higher variation between specimens. The same was found by Märdian et al. [6] and Elkins et al. [8]. They demonstrated that when increasing the WL, the total motion at the fracture site was increased, which is primarily through a dramatic and disproportionate increase in shear in relation to longitudinal motion. Elkins et al. [8] also found a WL threshold for titanium plates of 80 mm, where shear movement increased dramatically in comparison to axial movement and started to inhibit healing.

Experimental studies about shear movement have shown contradicting results. Augat et al. [12] showed using sheep tibia that axial compression/strain promotes periosteal callus and maturation, whereas shear movement has been shown to inhibit vascularization of the callus, resulting in lengthened healing and decreased mechanical stability. Lienau et al. [13] also showed using sheep tibia that a larger interfragmentary shear was associated with a reduced initial blood supply and resulted in less optimal healing path. They also found a greater fraction of fibrous tissue and mineralized bone. There was a lower stiffness of the callus in the group with greater shear movement, and that shear was responsible for the initial delay in healing. Steiner et al. [14], on the other hand, concluded in their study, also on sheep tibia, that optimized moderate axial stiffness together with certain shear rigidity is essential for enhanced fracture healing. Furthermore, inhibiting the influences of one loading direction can be compensated for by adjusting the other directional stiffness appropriately.

Harvin et al. [15] found no significant difference in union rates between fracture constructs with longer versus shorter WLs. They concluded that the understanding of how WL, plate stiffness, and secondary fracture healing are associated is more complex, but it appears that increasing the plate WL does not reliably increase (or decrease) union rates.

Limitations

Our study was not designed to identify the clinical significance of increasing WL. Our aim was to quantify the IFM and shear movement. During 50,000 cycles, the construct did not deform beyond the primary deformation, and the construct did not break under physiological load. It correlates well with the study by Henderson et al. [16], where implant failure occurred in 75% of cases after three months and 50% of cases at six months, with a non-union/delayed union. It is likely that metal fatigue of the plate is responsible for most plate failures and not the strength of the plate.

We did not use real bone but 4th-generation composite sawbones. The reason for using surrogate bone femora instead of cadaver specimens was to eliminate variations between specimens and thereby isolate the influence of the change in WL on IFM. Gardner et al. [17] compared 4th-generation sawbones with anatomical species and found them appropriate and similar to in vitro testing in all simulations examined.

In our study design, it was only possible to test with axial compression in the Instron machine, thereby eliminating bending and torsion. Also, we could account for the effect of ligaments, musculotendinous movement, and force. In the studies by Duda et al. [18] and Taylor et al. [19], the authors found that the loading of the femur in the weight-bearing standing phase was predominantly compression with very small bending forces and almost no torsion. We could therefore assume that the setup in our study was close to the normal force distribution in the stand phase.

While we did not find a significant difference in IFM between the two WL groups, we did find a notable difference in lateral shear movement. The clinical significance of increased shear stress remains to be elucidated, but it has been hypothesized that high shear stress can lead to transverse motion between fracture fragments, prolonging healing time and contributing to nonunion or delayed union [20]. The optimal level of IFM for the healing of femoral fractures has not been identified. Quantifying the relationship between IFM, strain, and healing rate remains an active area of research, aiming to mathematically describe these cause-and-effect relationships. Fracture healing depends on more than just WL, but also bone quality, bone loss, patient physiology, and proper reduction/alignment of the fracture. The 2-10% strain “rule” was proposed by Perren [1] to describe the supposed tolerance limits of fracture gap strain for successful bone healing. Below 2% gap strain (gap closure/original gap length), theoretically, there will be insufficient stimulation to form callus, and above 10% gap strain, excessive motion inhibits osteogenesis and predisposes a fracture to nonunion. Subsequent studies have challenged this rule, and likely, more than 10% strain can be tolerated for sufficient healing [21]. Considering this, we hypothesize that a difference of 0.8 mm in IFM will not affect the rate of fracture healing in a clinical setting.

Our study has the advantage of a quasi-physiological loading in the stand phase corresponding to approximately one body weight. If we had tested in the normal walking phase, the peak force should have been approximately 2-2.5 times higher [22,23]. After femoral fracture surgery, one may assume that load distribution on the fractured leg would be limited in the early phase of recovery, and therefore, the 750 N we selected would closely represent the load distribution in the early weeks after surgery.

Only plates made of stainless steel were tested in this setup, which are stiffer than geometrically equivalent plates made of titanium [8-10]. We might have found a different movement pattern if tested on titanium plates. Studies that looked at callus formation showed more callus in fractures treated with titanium plates compared with stainless steel plates [24,25]. Kandemir et al. [10] suggest that in simple fractures, with anatomical reduction, steel might be preferred because of less movement, and in fractures with comminution, titanium might be preferred because of greater movement and more micromotion between fragments.

## Conclusions

During an approximated physiological loading, we found less than 1 mm difference in axial IFM between a short and long WL. It is our estimate that this difference is not of clinical importance in terms of fracture healing. We found substantially higher levels of shear movement in the long WL, which we hypothesize may influence the fatigue strength of the implant, although this needs further investigation. We have provided a simple model to predict IFM based on WL and femur characteristics. The clinical impact of these findings should be investigated in clinical studies.
